# Hospital Meetings, &c.

**Published:** 1900-07-21

**Authors:** 


### HOSPITAL MEETINGS, &C.

### ST. GEORGE'S HOSPITAL.

A quarterly general Court of Governors of St. George's Hospital was held on Friday last, 13th inst., Mr. H. M. McPherson presiding, the other Governors present being Mr. Timothy Holmes (treasurer), Sir Henry Burdett, Deputy Surgeon-General Penny, Dr. Duka, Mr. A. Heathcote Long, Mr. R. Thorp, Mr. W. L. Barker, Mr. F. E. Villiers, Mr. Arthur Liddell, Mr. G. R. Turner, Mr. W. H. Bennett, and Dr. Rolleston.

The minutes of the weekly boards since the last quarterly court were read and confirmed; six new Governors were elected; certificates were granted to Mr. Percival and Mr. Winton, the outgoing house physicians; and other routine business transacted. An alteration was made in the law relating to quarterly and special courts, enabling that in May to be held on the date considered most convenient.

It was then moved by the Treasurer (Mr. Timothy Holmes) to empower the Weekly Board to sell stock to the amount of £4,000 to provide for current expenditure. This step would be necessary, he said, unless some very large donation or legacy were received in the very near future—of which there seemed at the moment no probability—as almost that amount would have to be expended on contracts now in hand in connection with the building completion. The power would, of course, only be exercised if it were necessary to do so, and they would only be too glad were someone to come along with the amount in his pocket and save them the necessity. They had not been quite unanimous at the last court with regard to the expenditure incurred. Mr. Shaw-Stewart considered it extravagant, and expressed himself in rather strong terms regarding it. The deficiency shown was partly due to the fact that last year they received very little in respect of legacies; and while their extraordinary income was thus lower than the average, their extraordinary expenditure had been going on in consequence of the alterations. There could be no doubt but that the result of the most recent of these was to add very greatly to the convenience and comfort of all the servants of the hospital, who were now lodged in their new quarters. But during the year, taking normal and extraordinary expenditure together, they had overrun the constable to the extent of £15,000.

Sir Henry Burdett seconded the motion. As one of the governors he had great pleasure, he said, in attending the meeting that day, though his numerous occupations did not often permit him to be present. But it had become a public duty for those who made a study of great public and philanthropic questions, and had knowledge of them to come forward and support such institutions as that. He had been over that hospital in successive years, as a visitor of the Prince of Wales's Fund, and it was within his knowledge exactly what had been done with the money expended on their reconstruction and alterations. This was an age of criticism. At their last quarterly meeting Mr. Shaw Stewart, who was now dead and whom he referred to with all respect, spoke adversely of their expenditure. But he spoke from the point of view of a period which had passed and which would not return.

### Hospitals in South Africa.

Hospital work to-day occupied so large a portion of the public mind that we had been threatened with what was almost a political crisis arising out of hospital affairs in South Africa, and the criticism applied to them. He was out of the country when that criticism appeared, or he should have felt compelled to point out that it was confined to too narrow a basis to warrant the attention it had received. If they turned to the letter of Mr. Burdett Coutts in the *Times* of June 27th they would see that he referred, not to one complete hospital even, but to the remnant of a hospital—a field hospital—at Bloemfontein; and his statements were confined to this relatively very small part of the whole system of military hospitals in South Africa. Although Mr. Burdett-Coutts made several visits he never attempted, so far as appeared—there was not one word in his letter to show that he did so—to ascertain whether the patients he saw on his later visits were the same that he had seen at the first. They who were familiar with hospital work knew that in campaigns and in civil life it was one of the necessities of great epidemics—such, for instance, as that at Maidstone—that they must have a receiving house for cases, from which they could be sent to the properly-constituted hospital. The institution at Bloemfontein never was, and was never meant to be a fully-equipped hospital—it was merely a receiving station for patients. His own impression was that the statements made by Sir Wm. MacCormac and Mr. Treves, than whom no two men were better entitled to speak on the subject, were not only true in fact, but might be taken as accurately representing the general state of hospital matters in South Africa. Mr. Burdett-Coutts seemed to be terribly shocked that patients should have been wrapped in blankets and placed on macintoshes and oilskins on the ground. At a receiving station such as that was beds were quite impossible, and they would bear him out in saying that had they been possible they would have been wholly undesirable under the circumstances. Some few days after the appearance of the strictures in the *Times* he had received a letter from a nursing sister in No. 6 general hospital at Naauwpoort. She wrote: "On the first day I cabled to you, we slept on the floor that night as our beds were packed up, and we were so stiff next morning we felt as if we had been beaten all over. I can quite understand how the 'Tommies,' after long exposure and no beds, say they cannot sleep the first nights they come into hospital because the beds are *too soft*." That was a good practical point, and a point they ought to make in favour of their confrères who were attending to the sick and wounded in South Africa. It was a first essential of criticism of hospital affairs that those who addressed themselves to it should have accurate practical knowledge of the circumstances and matters of which they treated.

The Public's View of Hospital Expenditure.

At their last meeting some severe criticisms had been passed regarding the expenditure at that hospital, and it was said it was so great that if it went on the public would soon cease to subscribe, and the hospitals would have to be taken over by the Government. He ventured to say that that was the exact opposite of the truth. If there was one thing the philanthropic British public wanted to-day it was an intelligent and a courageous administration of their great charities. It was true that there never was so large a sum spent on hospitals as was now the case, but it was also true that there never was a period when the volume of contributions voluntarily given by all classes of the population was so great as at present, and especially was this true of the metropolis. If they took ten years' growth they found an increase of 800,000 in the population of London, during which period they had had an increase of 400,000 in the number of patients at the London hospitals. At the same time, the contributions of the public had increased by £200,000, so that 10s. had been subscribed for each additional patient; and when they considered that a large proportion of the additional patients were out-patients, he thought they were entitled to say that the public had come forward to meet the increased obligation. What the hospitals were still suffering from was the deficiency of £100,000, which existed ten years ago between revenue and expenditure, and had never yet been wiped off. It was the object of the Prince of Wales's Fund, of the Hospital Sunday Fund, and the Hospital Saturday Fund to so raise the amount subscribedas to equalise income and expenditure.

The Position of a Great Hospital.

A great hospital like St. George's, if it was to keep the confidence of the public, must advance with the age, and must spend money not only on administration but on buildings. He thought the strongest testimony they had to a claim on the public was that they had spent this £100,000 on reconstruction and renewals, and so placed St. George's in a thorough state of efficiency. Its sanitary condition was now sound; its means of isolating infectious cases were the second to none; while its arrangements for the staff and the accommodation for its trained nurses were a credit to the institution. They were more than justified by the state of the buildings for the money that had been spent. The present condition of the finances was not satisfactory. If they included legacies in ordinary income—a large concession—they still had to face a deficit of £7,000 per annum, which they had to make good by realising their reserve. That was a state of things which ought not to continue to exist. If there was one hospital in London which should not be so situated, surely it was St. George's. He had been told by people connected with the Press and by others, "Why, St. George's has the finest site in London! Wealthy people cannot help seeing it all day long. It surely cannot be in want of money?" He believed that lay at the root of the difficulty. When they came to analyse the figures they were not creditable to the wealthy and influential people who lived all round them in this rich neighbourhood. The annual subscriptions used to be the largest, but they had been gradually falling back. From 1873 to 1883 the annual subscriptions averaged £8,200. From 1883 to 1893 they were £7,186, while in 1899 they were only £5,948 a-year.

The Road to Financial Success.

He would not have felt justified in speaking as he had done if he had not a suggestion to make with regard to the difficulty. It was always good to learn from one's neighbours. When Guy's Hospital was in a like position they exerted their strength and achieved a wonderful result. With the London Hospital it was the same, and at Charing Cross similar results were being produced by a similar effort. Lastly—and he would remind them of this because it seemed peculiarly applicable to their case—the old Great Northern Hospital had had the courage to move out from its old surroundings to the centre of North London, and had succeeded in ten years in raising the £100,000 expended on new buildings, and now had a larger income than ever, raised mainly in the surrounding neighbourhood. He rejoiced that they at St. George's were conservative in their methods, but at the same time he thought they had a special, pressing, and unanswerable claim upon the people of London for a proper share of support. They had never had festival dinners; he confessed he was not in love with festival dinners himself. But he thought if they were to ask for the assistance of a member of the Royal Family, who should be invited to place himself at their head and so enable them to let Londoners, and especially those living in their own district, know their needs, they would not only get the £10,000 a-year extra that they required, but should also be able to restore to their reserve fund the £100,000 they had so wisely spent upon reconstruction and new buildings. They could not blame their neighbours if they did not ask for what was needed, and show them where they could help. He advocated the appointment of a small committee to consider the subject with a view to taking steps as soon as the conditions were more favourable than they were to-day. He was sure the public would come forward and help one of the oldest and most reputable of their hospitals with both hands.

An Example to be Followed.

It was because he believed that the criticism levelled at their treasurer was not justified that he felt the hospital must be supported. He hoped that the example of Mr. Timothy Holmes, in coming forward at the conclusion of his professional services, to serve the hospital in an administrative capacity as its treasurer, might be more generally followed by members of the medical profession. His belief in the results to be obtained from a special effort was based upon accurate knowledge of the work of the hospital and also of the public who contribute. He hoped that they would unite and see what could be done toward making a sustained effort at the right moment, so that St. George's Hospital might in future have a greater, and not a less, share of the subscriptions of the public. (Cheers.)

The Prince's Fund and the Hospitals.

Replying to an observation by Mr. Villiers that the public had withheld subscriptions owing to a remark in the report of the Prince of Wales's Fund giving the idea that St. George's Hospital was not in need of money, Sir Henry Burdett said this statement was due to an entire misunderstanding which he hoped his presence there that day would help to remove. The committee of the Fund quite recognised that the income of St. George's was £10,000 behind what it ought to be; but they had a reserve fund, and there were many hospitals without one. They felt that they must first cope with the necessities of those whose needs were greatest. And they also felt that though St. George's wanted money, it was so obviously deserving of support that if the authorities would do what other hospitals had done and make a special effort they would soon be out of need. He should like it to go forth that the Prince's Fund considered that the fact that many of the patients at St. George's were servants from houses in the district gave the hospital a peculiar claim on that great and wealthy district, and noted with regret that its annual subscriptions were falling off. He fully recognised their claim on London for this extra £10,000 a year, and he would, with others associated with him, gladly render all the assistance in their power if the St. George's authorities would make the effort. As to the statement that had been made that people made a contribution to the Prince's Fund the excuse for striking off larger contributions to individual institutions, they had done their best to trace such cases, and he was bound to say had found them very few where traced. A little argument soon redressed the loss. Indeed, the fact that the total of public contributions had increased by £200,000 within the last ten years went to negative the idea that these cases were numerous or anything more than exceptional personal idiosyncracies of the few for which there was no accounting.

Mr. Timothy Holmes said the suggestion that had been made should certainly have the most careful attention of the authorities. They would give it their earnest consideration, and he had no doubt at all that something would be done.

The motion was adopted, and the proceedings terminated.

### CITY OF LONDON HOSPITAL FOR DISEASES OF THE CHEST.

### Unveiling a Bust of the Queen.

In spite of the fact that the thermometer registered 92 degrees in the shade on Monday, a large number of guests were present at the ceremony of unveiling a bust of the Queen, presented to the Chest Hospital, Victoria Park, by Sir M. M. Bhownaggree, K.C.I.E., M.P. The Lord Mayor, who was accompanied by the Lady Mayoress, came to carry out the ceremony, and was received by the treasurer of the hospital, Sir Edward Sassoon and Lady Sassoon, and by a guard of honour of the London Rifle Brigade. The bust, which was executed by Mr. A. E. L. Rost, stands just within the railings which divide the hospital grounds from the road, and here the visitors gathered, having first been refreshed by welcome tea in the cool corridors of the hospital. Speeches were brief; it was several degrees too hot for talking. Sir M. Bhownaggree offered his gift for acceptance, Sir Alfred Newton unveiled the bust, and the National Anthem was sung by Miss Maud Percival Allen and the children of Dr. Stephenson's Home. On behalf of the Committee of Management, Mr. Joseph Benson accepted the gift with becoming gratitude, and incidentally gave some interesting reminiscences of the hospital, which, he reminded his audience, stood in what had been Bishop Bonner's garden. Later, the Lord Mayor formally opened the newly-added balconies for "open-air" treatment, and the visitors found their way into the wards and talked to those patients not well enough to be out, while Dr. Stephenson's boys and girls sang their best, and the band of the Rifle Brigade played in the gardens. The City of London Hospital, known perhaps better in its immediate neighbourhood as the "Victoria Park Hospital," does a good work in the midst of a densely-populated district, and it is a pity that "West-enders" do not more often find their way thither. There are many things the hospital wants to aid it in its work; many additions to the wards which would make them brighter; new bedsteads, new floors, new furniture, and those who have a little money to spend might well and fitly hand it over to the treasurer of the City of London Hospital.

